# Dieting for Health

**Published:** 1855-05

**Authors:** 


### DIETING FOR HEALTH.

A man may diet as well as physic himself to death. Some time since a young man called to see me, thin, pale, despondent, and with a great variety of symptoms. On inquiry, I found he had been reading about diet, vegetable food and other similar subjects, and concluding that as many persons owed their ill health to over eating, he would eat very little of any thing, discarded meat of all kinds, and considered tea and coffee as decidedly poisonous in their ultimate effects. By this means, provisions being high, he concluded he would save money and health too. He had for some time been living on bread and potatoes, a small daily allowance, with as much cold water as he could possibly swallow, the object of that being to keep himself washed out clean. No wonder that such a man was an invalid—mind and body full of symptoms. "Dieting" is not starvation, it is living on substantial nourishing food, in amount sufficient to satisfy the wants of the system. A man is in little danger of eating too much, if he will confine himself to two or three plain articles of diet at any one meal; this is a secret which every man and woman in the land ought to know. Living exclusively on cold food will soon engender disease, especially in cold weather. And as certainly will a scant diet do the same if persevered in. A striking illustration of this is found in the history of one of the greatest men of modern times.

Napoleon the First, while a subaltern, was in such extreme poverty in Paris, that he was sometimes not able to raise ten cents with which to purchase a scanty dinner, and consequently had to go without any; he had even to borrow worn clothes from acquaintances, and to go out alternately with his brother, in the same coat. His food was so scanty that his face became pinched, harsh and angular; at length the skin became so diseased that it almost filled one with disgust to look at it, and it required all the skill of that eminent and able practitioner Corvisart, for several years to eradicate it.

Lesson 1.—*Disease will as certainly be engendered by too little food as by too much.*

2.—*Dieting consists in adapting the food in quantity as well as quality, to the wants of the system.*

